# Recent Threat of Scrub Typhus in India: A Narrative Review

**DOI:** 10.7759/cureus.30092

**Published:** 2022-10-09

**Authors:** Vaibhav B Kore, Shital M Mahajan

**Affiliations:** 1 Microbiology, Jawaharlal Nehru Medical College, Datta Meghe Institute of Medical Sciences (Deemed to be University), Wardha, IND

**Keywords:** zoonotic diseases, threat, immunity, eschar, india, scrub typhus

## Abstract

Scrub typhus is an endemic illness transmitted by vectors and induced by bacteria. It is the most common and severe rickettsial disease. There are many more cases every year with a significant case fatality rate. Despite being a serious public health threat in India, it is uncertain how widespread and burdensome scrub typhus is. The scarcity of statistical information and pertinent health records on scrub typhus in the outbreak region demonstrates that there is still a significant knowledge gap about this neglected illness. Clinical manifestations of this illness include kidney failure, disability, and severe kidney failure. Undifferentiated symptoms, late diagnosis, and treatment failure are all responsible for deaths. Knowing about this disease is important from a public health point of view due to difficulties in specific diagnosis and a shortage of laboratory services in so many places. India is known to have scrub typhus cases, and doctors should be aware of this potentially dangerous but easily curable illness. The disease is highly difficult to identify clinically from other acute afebrile infections due to common symptoms and a paucity of the lesion in the Indian population. The mainstay of diagnosis is antibody-based serological testing. Within the first week of symptoms, scrub typhus can be diagnosed using molecular and serological tests. Our objective is to identify how severe scrub typhus is in India and to investigate the current epidemiology, etiology, complications, management, and treatment of the disease in both long-established endemic regions and new infection foci.

## Introduction and background

**Figure 1:**
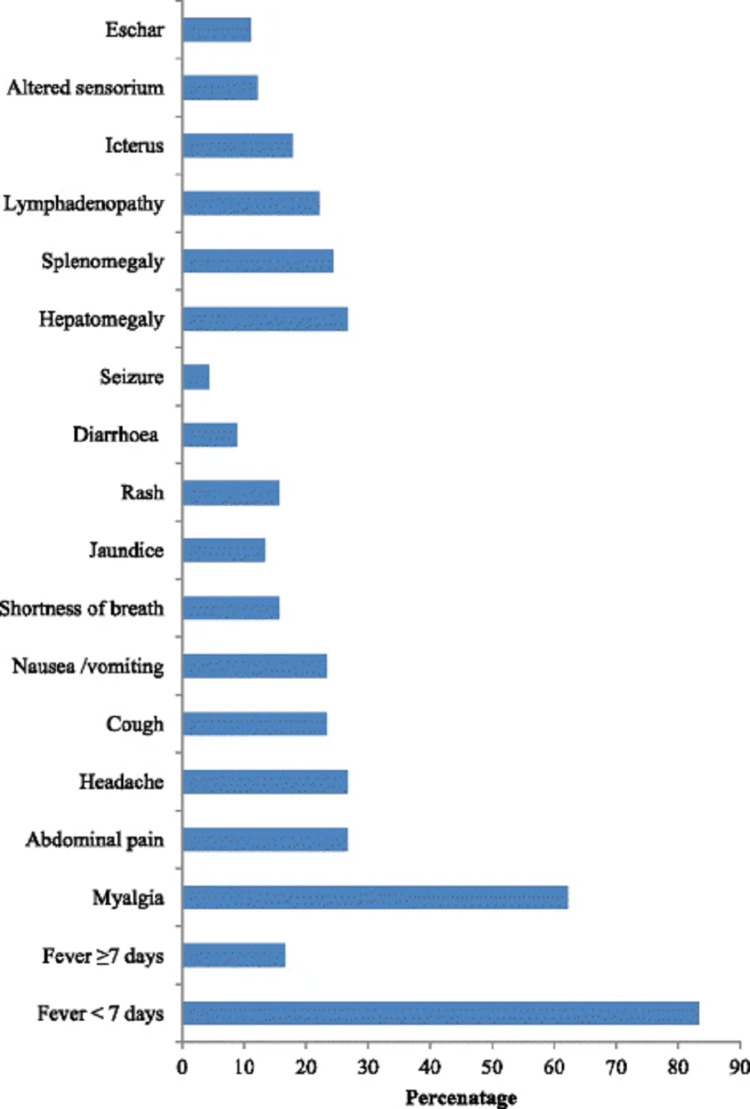
Clinical symptoms and signs among hospitalized patients with scrub typhus in Meghalaya, India. Source: [3]

*Orientia tsutsugamushi* is a tiny obligate intracellular, gram-negative bacterium that causes acute febrile fever, also known as tsutsugamushi disease. Its polysaccharides are antigenically identical to the proteus OX-K, used in serologic assays to diagnose the disease. *Orientia tsutsugamushi* is spread to people by biting an almost tiny, frequently colored larva of trombiculid mites (chiggers) (red). During the rainy season, infected chiggers are more frequently found in densely vegetated areas, which is why the condition is also known as river or flood fever. Mites lay their eggs from July to December, on average. Even though rodents and mites may be found in other areas, the word "scrub" was adopted due to the general vegetation that promotes the chigger-host connection. The disease is found only in certain global places [1]. It mainly causes eschar at the biting site, developing into a black crust-like structure and a cigarette bundle. In recent years, scrub typhus has spread throughout India and has emerged as a significant cause of severe febrile illness, and has a higher death rate and fatality rate. Several states in India have reported high numbers of cases of this disease: in the south, Tamil Nadu, Andhra Pradesh, Karnataka, and Kerala; in the north, Himachal Pradesh, Uttaranchal, Jammu, and Kashmir; in the north-east, Meghalaya, Assam, and Nagaland; in the east, West Bengal and Bihar; and in the west, Maharashtra and Rajasthan (Figure 1) [2].

Uncertain estimates of scrub typhus mortality in the present day range from 40% to 45% for cases not treated with antibiotics. However, it can be challenging to determine the actual mortality because its severity is considered to vary depending on geographical strains, infectious dosage, patient age, and comorbidities [4]. Acute respiratory distress syndrome (ARDS), myocarditis, acute renal damage, meningoencephalitis, and disseminated intravascular coagulation are all consequences of scrub typhus. Most of the complications of scrub typhus are organ-specific [5]. Scrub typhus became an epidemic in West Bengal and Assam during the Second World War. The epidemic form spread progressively over most of India. Scrub typhus is an indigenous disease in some parts of Asia, including the eastern and western regions [6].

## Review

Epidemiology and distribution

Scrub typhus affects about one billion people worldwide, and there are thought to be one million new cases yearly [7]. It may be found in India's Shivalik mountains, which stretch from Assam to Kashmir, the Eastern and Western Hills, and the Vindhyachal and Satpura mountains in the country's central area. Disease outbreaks were recorded in Himachal Pradesh, Tibet, and Darjeeling (West Bengal) in 2003-2004 and 2007. This illness is also frequently seen in areas with sandy beaches, alpine deserts, and tropical rain jungles. The ideal environment for developing infectious vectors can be found in specific niches such as grassy areas, riverbanks, and forest clearings. These restricted places, often known as scrub typhus islands, are high-risk for human populations [5]. Scrub typhus epidemiology is hampered by bacterial strains' antigenic and molecular diversity and their unclear relationship with human pathogenicity [8]. Efforts to prevent and control scrub typhus will aid in a better understanding of the disease's epidemiology [9].

Agent factors

*Rickettsiae* are gram-negative, non-motile, non-capsulated, and pleomorphic bacterium. They are cultivated in the yolk sac of the chick embryo. They are coccobacilli that occur in pairs or chains. *Rickettsia tsutsugamushi* is the disease-causing agent in India. Compared to other rickettsial diseases, it has a different antigenic structure. *Leptotrobidium* delineates and *L. akamushi* serves as the reservoir for scrub typhus. Chiggers are insects in the larval stage. The larval stage takes the blood of the host. Nymphal and adult forms travel peacefully in the environment. The majority of Asia is home to these endemic creatures. The infectious mites, sometimes called "chiggers," are the means of disease transmission. Blood meals are only consumed during the larval stage [10]. The disease is transmitted by the bites of infected arthropod vector mites. These mites take their hosts' lymphatic and body secretions. They keep the disease throughout their entire existence, and it later spreads to their eggs; this process is known as transovarial transmission. The transmission of an infection from an egg to a larva or adult is referred to as transstadial transmission. When the larvae feed, a significant portion of *O. tsutsugamushi* from their salivary glands is injected into their host [11].

Pathogenesis

The disease is generally spread to humans by the biting or feces of an infectious arthropod vector. The mite has four life forms: egg, nymph, larva, and adult. Only the second form is infective to humans. Bacteria penetrate vascular endothelial cells, causing diffuse vasculitis and microvascular ulcerations that activate cytokine, macrophage, and T cells, resulting in severe vascular bleeding and end-organ injury in organs such as the lungs, kidneys, and heart [12]. Activated cells result in a broad spectrum of inflammatory processes, with endothelium and non-endothelial cells generating several cytokines that can be both helpful (i.e., antimicrobial) and induce tissue death in the infected individual. This type of immunological response can cause serious problems such as infectious liver diseases, kidney failure, meningitis, encephalitis, and respiratory distress in the form of acute distress syndrome, as well as pericarditis in some instances [13].

Clinical features

*Orientia tsutsugamushi* typically takes 10-12 days for humans to develop symptoms of the illness; these include pyrexia, pain in the head, muscle pain, coughing, and gastric symptoms. Pyrexia of unexplained origin (PUO) is patients' most frequent presenting symptom [14]. The most typical symptoms are fever and headache. Several investigations found that between 95% and 100% of confirmed cases had fever [15,16]. Eschar development occurs when a chigger feeds on the host and is a hallmark sign of scrub typhus. Eschar develops into papules that eventually ulcerate, and there is a production of black crust-like growth. Eschars are typically seen on the front of a man's body close to a woman's umbilicus, neck, and chest. In addition to the previously indicated locations, children's axillae frequently contain eschars [17,18]. Patients with severe illnesses may develop meningitis or encephalitis, which can result in agitation, delirium, or even seizures. Although they are uncommon, focal neurological symptoms have been documented. Cerebrospinal fluid alterations resembling those seen in viral or tuberculous meningitis may be seen in laboratory tests of scrub typhus patients [19]. In a small percentage of cases, scrub typhus can induce hearing impairment, eventually resulting in hearing loss. Patients with scrub typhus have reported hemorrhages and coagulation-related issues, primarily gastrointestinal issues. Patients with severe secondary illnesses can develop ulcers, numerous erosions, and gastrointestinal mucosal bleeding [20]. Lymphadenopathy, conjunctivitis, hepatomegaly, and arthralgia may be seen in the heterogeneous strain types. Disease-affected area increases mortality. Therefore, effective control and prevention should be offered from the general public's perspective.

Diagnosis

The chief complaints of fever and eschar formation on the skin help diagnose scrub typhus, but eschar is variably present. Therefore, other investigations should be done to diagnose the disease. Serology is the primary investigation of choice for the diagnosis of scrub typhus.

Weil-Felix Test

The Weil-Felix OX-K agglutination reaction (WF test) is the simplest currently used test; it is cheap and simple to run, and results are available immediately; nevertheless, it has more specificity than sensitivity [21]. WF test depends on identifying agglutinin to different Proteus strains that respond to agglutinogen from *Rickettsia* species when they cross-react. In the primary infection, at the end of the first week, a significant antibody titer is mainly IgM type, whereas IgG titer is seen at the end of the second week. Scrub typhus is detected by the OXK carbohydrate antigen of Proteus mirabilis. In underdeveloped countries, WF is the most widely utilized serological test. The false-positive report may obtain in typhoid and other diseases. WF's negative test cannot eliminate typhus disease. The gold standard method of detection is indirect immunofluorescence antibody (IFA). Before their seroconversion, this test can confirm the diagnosis. IFA is very expensive, but the result is available within hours. Another rapid diagnostic test that detects antibody levels is an immunochromatographic test (ICT). The Western immunoblot test using sodium dodecyl sulfate-gel electrophoresed and electroblotted is a powerful and accurate serodiagnosis technique for seroepidemiology, and verification of serologic findings agglutinogen is suitable for large-scale monitoring. It also aids in the investigation of cross-reactive strains [5]. IFA typically employs antigens from three serotypes: Karp, Kato, and Gilliam. Nonetheless, considerable antigenic diversity has been discovered everywhere it has been investigated [22]. The recombinant protein-based ELISA test is essential for identifying the different strains of disease-causing bacteria.

Culture

The different culture techniques include Vero cell culture, embryonated chicken yolk sacs, and L929. A buffy coat of anticoagulated blood, triturated clot, serum, necropsy tissues, skin biopsies, and defibrinated blood culture is used to get the sample. Vero or L929 cells have enabled efficient and quicker Rickettsiae identification, but HEL or MRC5 cells hinder contact inhibition [23]. For isolation of bacteria, cell culture takes a minimum of four weeks. Bacteria is cultured at 35-degree calcium temperature in a 5% CO2 environment [24]. The cells were collected, pelleted, and kept at 80°C. After the cytopathic plaque formation was achieved, 90-100% of cells were plated of the entire monolayer.

Molecular Test

The polymerase chain reaction is used to detect the genetic structure of bacteria. The collected samples are from lymph nodes, skin biopsy, and eschar. Real-time PCR tests based on GroEL are more sensitive and provide a more quantitative result [25]. Loop isothermal amplification technology is also helpful in the diagnosis of scrub typhus. The nested PCR technique detects deoxyribonucleic acid before the antibody development.

There could be leukocytosis and thrombocytopenia. Between 75% and 95% of cases may have elevated transaminases. Hyperbilirubinemia and hypoalbuminemia are common conditions. Creatinine increases may occur in extreme situations. Enlargement of the liver and spleen can be seen using ultrasound. On chest X-ray, there may be bilateral infiltrates and pleural effusion. Other differential diagnoses that should be eliminated include dengue, malaria, and pyrexia of unknown origin. Indirect fluorescent antibody and serology are the specific and commonly used methods for diagnosing scrub typhus.

Management

Rapid antibiotic treatment decreases the duration of the illness, minimizes the likelihood of consequences, and ultimately minimizes mortality and death from scrub typhus infections. Tetracycline is the most commonly used drug for scrub typhus, with doxycycline being the treatment of choice. Doxycycline (2.2 mg/kg/dose twice PO or IV, maximum 200 mg/day for 7-15 days) and tetracycline (25-50 mg/kg/day split every 6 hours PO, maximum two g/day per mouth, duration 7-15 days) are the approved treatments for scrub typhus. Tetracycline 200 mg might be used as a single dose for prophylaxis. However, findings of excellent resistance make it challenging to select effective antibiotics [26]. Chloramphenicol (500 mg QID orally for 7-15 days for adults) (50-100 mg/kg/day split every 6 hours IV, maximum three g/24 hours) is one option for alternative regimens. Chloramphenicol should be monitored if it is administered to keep serum concentrations between 10 and 30 g/mL. Treatment should be continued for at least five days until the individual has been afebrile for at least three to four days to prevent a recurrence. Reduced doses of chloramphenicol drug should be used in cases of hepatic impairment and avoided during pregnancy. Azithromycin (500 mg used daily for three days), rifampicin, and roxithromycin are the other antibiotics that have been proven to be beneficial. Rifampicin is more effective and better than doxycycline in treating scrub typhus. According to studies, roxithromycin (150 mg twice a day) is just as beneficial as doxycycline or chloramphenicol, implying that it might be used as a substitute treatment for children or pregnant women. Chemoprophylaxis with doxycycline (200 mg once a week) can halt the illness for short periods and prevent a recurrence. Doxycycline therapy for scrub typhus has shown promising results when started before infection. Drug resistance to scrub typhus has been found in some countries. Patients here need treatment with rifampicin.

Scrub typhus in the risk group

Scrub Typhus in Pregnancy

When mothers have an acute febrile illness while pregnant, there are more chances that the disease can spread to the fetus through the placenta and parturition when blood mix with the neonates. Neonatal scrub typhus may happen during perinatal care through a blood-born route [27]. Preterm birth, a rise in fetal loss, and small for gestational age newborns are possible correlations [28]. Chloramphenicol can pass through the placenta and should be given carefully to stop fetal transmission when used in the third trimester of pregnancy. It is placed in category C medication [29]. Doxycycline, a category D medication, should not be used by pregnant women. In a reported retrospective cohort series, Poomalar et al. reviewed eight cases of typhus disease in pregnancy for symptomatology, comorbidities, and mother and neonatal outcomes [30].

Scrub Typhus in Childhood

Children with scrub typhus typically only experience mild-to-moderate symptoms. Most kids complain primarily of lymphadenopathy, hepatomegaly, and gastrointestinal issues. The case fatality rate is modest. Compared to adults, children are less likely to die or experience complications.

Scrub Typhus in India

For the past 20 years, the disease has been rising in India. In 2010, 2011, and 2012, scrub typhus infections were routinely recorded by the Integrated Disease Control Programme's West Bengal State Surveillance unit in Kurseong, Mirik (West Bengal's Darjeeling district). However, no fatalities were recorded, and doxycycline was used to treat every case with satisfactory results. Disease outbreaks were observed in some of the west Bengal districts up to the 1960s, making it historically one of the country's scrub typhus-endemic regions. There were no recorded outbreaks for a very long time, which lasted until 2005. A scrub typhus outbreak happened in the Darjeeling area of West Bengal in 2005 and was intensively researched epidemiologically after that [31].

Preventive measures

Vector control should be done to avoid accidental infection. Vegetables where rats live should be cleaned, and insecticide should be used on the ground. To prevent rickettsial infections, lice, mites, and other vectors need to be controlled with the proper use of an insecticide. For personal prophylaxis, clothes and blanket should be impregnated with benzyl benzoate, and mite repellent (diethyltoluamide) should be used on exposed skin surfaces.

Vaccines

Since human studies did not duplicate the success of animal studies, the first attempts to develop vaccines using dead *O. tsutsugamushi* were unsuccessful. The variety of strains, volunteers' inability to tolerate live vaccines because there are not any naturally attenuated species of the scrub typhus, and the failure to produce long-lasting cross-protection even with eradicated serotypes have all been put out as explanations for this. Many more vaccine formulations have been investigated for immunization, including the killed, live, antimicrobial, attenuated, and subunit vaccinations. The lethal vaccines were created utilizing formalin-treated variants such as the Karp and Gilliam strains. According to the studies of Smadel et al., most individuals who got a live vaccination followed by an antimicrobial therapy obtained protective immune responses against the identical strain that lasted about a year [32]. A successfully attenuated Karp strain of O. tsutsugamushi was created through gamma irradiation [33]. Despite having the ability to infect cells, this chemical did not cause illness.

Additionally, it was demonstrated that irradiated strains offered equivalent resistance for 12 months and cross-resistance for about half a year. Due to issues with producing, preserving, and standardizing the vaccine, the study on irradiation immunizations for scrub typhus was abandoned, and the attention switched to creating subunit immunizations. Subunit vaccinations have predominantly targeted 22, 58, 56,110-kDa, and 48 amino acid agglutinogens [34]. Attempts were also undertaken to create recombinant complexes to improve cell-mediated and humoral defense against scrub typhus [35]. It is necessary to further look into the 47-kDa antigen's potential as a scrub typhus vaccine candidate. Despite being a less common protein, the 110-kDa *O. tsutsugamushi* antigen has strain- and group-specific epitopes. Because humans can recognize this molecule after initial infection, it is a good candidate for scrub typhus immunization [36,37]. When autotransporter protein and OA56, a crucial outer membrane protein of *O. tsutsugamushi*, are present, the development of bacterial pathogenesis depends on ScaA, an autotransporter protein [38]. Immunity against heterologous strains and preventive immunity against deadly challenges with the same variant are both significantly increased by vaccination with the tsutsugamushi ScaA strain.

## Conclusions

Scrub typhus spreads faster in the country due to the resistance developed against different strains. Most infectious outbreaks are seen in some Tamil Nadu districts, Meghalaya, and Darjeeling. Scrub typhus is accompanied by high morbidity and death rate. Patients who present with fever during the monsoon season require a heightened index of suspicion. Higher disease risk is primarily seen in agriculture workers and pregnant patients. IgM ELISA test helps in the diagnosis of the disease. This review highlights the prompt diagnosis, treatment, and management of scrub typhus.
